# Editorial: Culture and emotion in educational dynamics, volume II

**DOI:** 10.3389/fpsyg.2025.1558664

**Published:** 2025-02-18

**Authors:** Enrique H. Riquelme, Silvia Cristina da Costa Dutra, Dario Paez

**Affiliations:** ^1^Diversidad y Educación Intercultural, Temuco Catholic University, Temuco, Chile; ^2^Departamento de Psicología y Sociología, University of Zaragoza, Zaragoza, Spain; ^3^Andres Bello University, Santiago, Chile

**Keywords:** culture, emotion, education, dynamics, students, family

This volume has focused on emotional dynamics in educational environments. In this editorial we offer a themed review of the fascinating and diverse contents of this Field of Investigation. This volume II can be organized into three blocks, the first of them associated with the socio-affective experience of teachers, the second associated with the family-school bond and the third directly linked to the affective dynamics of students of different levels of training.

- Socio-affective factors involved in the teaching experience, highlighting technology, emotions and work performance: Teaching is a profession that faces significant challenges, among which teacher burnout has become a topic of growing concern. In this context, the integration of educational technologies, such as the use of GPT (Generative Pre-trained Transformer), has been the subject of study to understand its impact on the teaching experience. The work of Chen et al. reveals that, although the integration of GPT does not have a significant direct effect on teacher burnout, the classroom climate plays a crucial mediating role. This implies that a positive climate can mitigate the stress faced by educators, suggesting that technology, if used appropriately, can contribute to creating a more favorable environment for teaching.

In the same line, the study by Lai et al. delves into how educators' emotions are shaped by their work context and personal characteristics. Through a phenomenological approach, it *is argued* that emotions are not only individual reactions, but are influenced by sociocultural factors and the environment in which teachers work. This theoretical framework suggests that in order to address teacher burnout, it is essential to consider not only technology, but also the emotional context in which educators operate. In this sense, the management of emotions in the classroom is a critical aspect that affects the work performance of teachers.

Hao's study examines how emotional labor impacts the performance of college educators. The findings indicate that shallow performance, which refers to acting superficially in the classroom, has a negative effect on relational and task performance. On the other hand, deep performance and proactive authenticity are positively correlated with performance in various areas. This suggests that teachers who manage their emotions effectively and who are authentically engaged in their work tend to perform better, which in turn can contribute to a more positive classroom environment.

The importance of socio-emotional skills in teaching is also highlighted by Sáez-Delgado et al.. Their study validates an instrument designed to measure teachers' socio-emotional skills, identifying four key dimensions: cognitive management of emotions, empathy, teacher-student relationship, and adverse classroom climate. The validation of this instrument provides a valuable tool to investigate and improve the socio-emotional competencies of educators, which is essential for their wellbeing and performance. The interrelationship between technology, emotions, and teachers' job performance suggests that a holistic approach is needed to address teacher burnout. The integration of technologies can offer opportunities to improve teaching, but its effectiveness depends largely on the cimate of the classroom and the emotional management of educators. In addition, the development of socio-emotional skills is essential for teachers to manage their emotions and establish positive relationships with students.

- Family-School bonds: relationship between family functionality and the emotional wellbeing of adolescents in the educational context has been the subject of growing interest in research. The study by Molina Moreno et al. addresses this issue by revealing how family functionality positively impacts essential social and emotional competencies, such as optimism, sociability and self-efficacy in adolescents in Spain.

This study establishes that a functional family not only contributes to improving the mental health of young people, but also acts as a partial mediator between humanization competencies and academic burnout. The research underscores the importance of family-centered interventions, suggesting that improving family functionality can foster greater psychological wellbeing and improved academic effectiveness.

In parallel, the study by Ratka-Pauler et al., explores how parents' beliefs about the acquisition of literacy influence their children's learning. The research is based on the theory of planned behavior and focuses on the parents' perspective, showing the importance of their attitudes toward joint literacy activities. The results suggest that parents are active agents in the acquisition of literacy skills, and that their beliefs about these activities may be a determining factor in their children's educational success.

Parental education anxiety also plays a crucial role in children's learning, *as* the study by Yin et al. *suggest, examining* the relationship between parental anxiety about child' education and learning anxiety in children. This study highlight how parenting style acts as a mediator and how extracurricular tutoring can moderate the relationship *between parents and children anxiety*. The data reveal an association between parents' educational anxiety and children's learning anxiety. Parenting styles that involve rejection and overprotection increase this Negative affectivity, while a style based on emotional warmth reduces it. In addition, academic tutoring is identified as an important moderator that can lessen the impact of parental anxiety on children's learning anxiety.

- Affective dynamics associated with the student experience: innovative methodologies and recognition of students' emotional needs play a crucial role in their academic success and overall wellbeing. In this sense, valuable perspectives are offered on how different approaches can influence the educational experience, from the use of immersive narratives to the impact of emotional factors on academic performance.

The article by Brunetti et al. presents an educational methodology based on interactive narratives that seek to develop competencies in students through emotional journeys. This methodology is integrated into curricular activities with the aim of achieving both curricular and cross-curricular learning objectives. The theoretical principles of immersive education are articulated around four key concepts: motivation, dramatic structure, self-involvement, and fostering ongoing engagement. These elements not only seek to attract students' attention, but also to manage their emotional reactions, which is essential for effective learning. Research suggests that by engaging students in narratives that resonate emotionally with them, their engagement and, consequently, their academic performance can be improved.

In the same *vein*, *Yin and Soon Ko's* study examines the effectiveness of group art therapy on the stress coping ability of Chinese students in South Korea. The results demonstrate that participants who attended the art therapy sessions showed a significant reduction in stress and an improvement in their coping skills compared to the control group. This therapeutic approach offers a promising avenue for providing emotional support to international students, helping them manage stress and better adapt to their new academic environment.

In addition, the study by Tassew Woreta addresses the factors that influence the academic engagement of high school students in Ethiopia. Through an integrative model, parental and peer academic socialization, self-efficacy and outcome expectations are identified as significant predictors of academic engagement. Research shows that positive relationships with parents and peers not only boost self-efficacy, but also support more optimistic outcome expectations, which, in turn, translates into greater academic engagement. This emphasizes the need for a supportive social environment that encourages students' active participation in their learning.

The study by Slimi et al. investigates i*mpostor* syndrome, a phenomenon that has increased among doctoral students and can have adverse effects on their mental health and academic career. This study examines the relationship between supervisors' empathy and the presence of this syndrome in PhD students in Tunisia. The results indicate a significant negative correlation: as supervisor empathy increases, the prevalence of impostor syndrome among students decreases. This underscores the importance of empathy in the academic environment, suggesting that supportive and supportive supervision can be a key factor in mitigating students' negative experiences, thereby improving their emotional wellbeing and academic performance.

Finally, the study by Soufi Amlashi et al. examines how acculturation stress affects the psychological health of international students. The systematic review reveals a moderate correlation between acculturation stress and negative psychological outcomes, such as depression and psychological distress. These findings highlight the importance of providing adequate psychological support to international students, who often face additional challenges in adapting to a new cultural environment.

*Globally*, these studies illustrate the interconnectedness between innovative educational methodologies and students' emotional wellbeing. Immersive education, empathy in supervision, academic socialization, acculturation stress management, and group therapy are aspects that, when addressed, can positively impact the educational experience. Implementing approaches that consider both academic and emotional aspects is essential to creating an educational environment that not only fosters learning, but also supports the holistic wellbeing of students.

